# Functional Reformulation of Shortbread Cookies with *Urtica dioica* L. Powder and Erythritol: Effects on Sensory Quality, Glycaemic Index and Glycaemic Load

**DOI:** 10.3390/nu18182973

**Published:** 2026-09-11

**Authors:** Ewa Raczkowska, Paulina Sławińska, Klaudia Woźniak, Karolina Rak, Robert Gajda, Sabina Lachowicz-Wiśniewska

**Affiliations:** 1 Department of Human Nutrition, Faculty of Biotechnology and Food Science, Wroclaw University of Environmental and Life Sciences, 37 Chelmonskiego Street, 51-630 Wroclaw, Poland; pslawinska.dietetyk@gmail.com (P.S.); karolina.rak@upwr.edu.pl (K.R.); robert.gajda@upwr.edu.pl (R.G.); 2 Department of Nutrition and Food, University of Kalisz, Pl. W. Bogusławskiego 2, 62-800 Kalisz, Poland; s.lachowicz-wisniewska@uniwersytetkaliski.edu.pl; 3 Department of Biotechnology and Food Analysis, Wroclaw University of Economics and Business, Komandorska 118/120 Street, 53-345 Wroclaw, Poland

**Keywords:** *Urtica dioica* L., nettle powder, erythritol, shortbread cookies, glycaemic index, glycaemic load, sensory evaluation, functional bakery products

## Abstract

**Background/Objectives:** Reformulation of bakery products with functional ingredients and reduced-sugar alternatives is an important strategy for improving nutritional quality and lowering postprandial glycaemic response. This study evaluated the effects of *Urtica dioica* L. powder incorporation and sucrose replacement with erythritol on the sensory properties, glycaemic index (GI) and glycaemic load (GL) of shortbread cookies. **Methods:** Cookies were prepared by replacing wheat flour with 0–50% nettle powder and using either sucrose or erythritol as a sweetener. Sensory evaluation was performed by 120 consumers using a 9-point hedonic scale. Based on sensory acceptance, cookies containing 0%, 10% and 20% nettle powder were selected for in vivo determination of GI and GL in 22 healthy adults according to ISO 26642:2010. Data were analysed using one- and two-way ANOVA. **Results:** Increasing nettle incorporation significantly reduced sensory acceptance, particularly for colour, taste, odor and overall acceptability, whereas cookies containing 10–20% nettle powder remained well accepted. Nettle addition significantly reduced GI in both sucrose- and erythritol-sweetened cookies, from 73.77 ± 11.05 to 52.10 ± 11.02 and from 68.69 ± 12.10 to 48.10 ± 9.03, respectively. The lowest GI (48.10 ± 9.03) was observed in cookies containing 20% nettle powder and erythritol. Erythritol did not significantly affect GI (*p* = 0.797), but significantly reduced GL compared with sucrose (*p* < 0.001). The lowest GL was observed for the 20% nettle-erythritol formulation (4.16 ± 0.80). No significant interaction between nettle powder addition and sweetener type was observed for either GI or GL. **Conclusions:** Incorporation of 10–20% nettle powder provided the most favourable balance between sensory acceptance and reduced glycaemic impact, while higher substitution levels substantially impaired sensory quality. Erythritol further reduced GL by lowering the amount of available carbohydrates in a standard serving, although it did not significantly affect GI under the carbohydrate-standardised conditions used for GI determination. The combination of moderate nettle enrichment and erythritol may therefore provide a lower-GI, lower-GL food option with acceptable sensory properties. However, these findings are based on a small cohort of healthy young adults and should not be interpreted as evidence of a therapeutic effect or directly extrapolated to populations with impaired glucose metabolism.

## 1. Introduction

Bakery products are an important component of the human diet due to their wide availability, affordability and high consumer acceptance; however, shortbread cookies are typically rich in refined wheat flour, saturated fatty acids and sucrose, resulting in relatively low nutritional value and a relatively high postprandial glycaemic response. Growing consumer interest in healthier diets has stimulated the development of bakery products enriched with functional ingredients to improve their nutritional quality while maintaining desirable sensory characteristics [1,2,3].

One of the most promising approaches for improving the nutritional quality of cereal-based products is the incorporation of plant materials naturally rich in dietary fibre and biologically active compounds. Stinging nettle (*Urtica dioica* L.), traditionally used in both nutrition and herbal medicine, has attracted increasing scientific attention because of its high concentrations of dietary fibre, chlorophylls, carotenoids, phenolic compounds, vitamins and essential minerals [4,5]. Numerous studies have demonstrated antioxidant, anti-inflammatory, antimicrobial and antidiabetic activities of nettle, indicating its considerable potential as a functional food ingredient [4,5,6].

Apart from its health-promoting properties, nettle may improve the nutritional profile of cereal products by increasing their fibre and micronutrient contents while simultaneously reducing starch digestibility. Maietti et al. (2021) demonstrated that the enrichment of bread-making products with *Urtica dioica* significantly increased antioxidant capacity and mineral content without adversely affecting technological quality at moderate incorporation levels [6]. Similarly, Krawęcka et al. (2021) reported that partial replacement of durum wheat semolina with nettle powder increased protein, ash and antioxidant compounds while reducing the estimated glycaemic index of pasta [7]. More recently, the incorporation of nettle leaf flour into gluten-free corn cookies was shown to enhance nutritional value and significantly decrease the estimated glycaemic index. However, increasing levels of nettle also resulted in darker colour and reduced sensory acceptance [8].

In parallel with the use of plant-derived functional ingredients, reducing added sugars has become an important strategy in bakery reformulation, as excessive free sugar consumption is associated with an increased risk of obesity, type 2 diabetes and cardiovascular diseases [9,10]. Erythritol is one of the most widely accepted sugar alcohols because it provides sweetness comparable to sucrose while contributing negligible energy and having virtually no effect on blood glucose or insulin concentrations. Consequently, erythritol has been increasingly incorporated into foods intended for individuals requiring glycaemic control [11,12].

The nutritional quality of carbohydrate-rich foods is commonly evaluated using the glycaemic index (GI) and glycaemic load (GL), which describe both the quality and physiological impact of dietary carbohydrates. The nutritional quality of carbohydrate-rich foods can be evaluated using the glycaemic index and glycaemic load, which reflect carbohydrate quality and the glycaemic impact of a food, respectively [13,14,15].

Raczkowska et al. (2024) reported that substitution of sucrose with erythritol together with fruit pomace significantly lowered the glycaemic index and glycaemic load of shortbread cookies while maintaining satisfactory sensory acceptance [2]. Despite the growing body of literature concerning nettle-enriched bakery products, almost all available studies have evaluated either technological properties or estimated glycaemic responses measured in vitro. To the best of our knowledge, studies simultaneously investigating the effect of nettle addition and erythritol substitution on the sensory quality together with in vivo glycaemic index and glycaemic load of conventional shortbread cookies are currently lacking.

Therefore, the aim of the present study was to evaluate the effect of replacing wheat flour with different levels of stinging nettle (*Urtica dioica* L.) powder and replacing sucrose with erythritol on the sensory properties, glycaemic index and glycaemic load of shortbread cookies. Sensory evaluation was performed using a 9-point hedonic scale, whereas GI was determined in vivo according to ISO 26642:2010. We hypothesised that moderate incorporation of nettle (10–20%) combined with erythritol would reduce the glycaemic response while maintaining acceptable sensory characteristics, providing a basis for the development of nutritionally improved bakery products.

## 2. Materials and Methods

### 2.1. Composition and Preparation of the Experimental Cookies

Commercial dried nettle leaf powder (*Urtica dioica* L.) (Diet-Food, Opatówek, Poland) was used, along with wheat flour (type 450), sucrose or erythritol, butter (82% fat), and egg yolks. All food ingredients were purchased from local retail stores. Wheat flour was partially replaced with nettle powder on a weight basis (*w*/*w*) at levels of 0, 10, 20, 30, 40 and 50%. The formulation of each cookie variant contained fixed proportions of sucrose/erythritol (15.2%), butter (30.3%), and egg yolks (9.1%), while the proportions of wheat flour and nettle powder varied according to the formulations presented in Table 1.

The cookie production process consisted of the following steps:

• Mixing of dry ingredients: Wheat flour, sucrose or erythritol, and nettle powder were mixed for 2 min using a KitchenAid mixer (model 5KPM5, KitchenAid, Springfield, OH, USA).
• Dough preparation: Butter and egg yolks were added to the dry ingredients, and the dough was mixed for 3 min using the same mixer.
• Chilling: The dough was refrigerated at 4 °C for 60 min.
• Sheeting and cutting: The chilled dough was rolled to a thickness of 0.5 cm and cut into circular cookies with a diameter of 5.0 cm.
• Baking: The cookies were baked at 180 °C for 8 min in a convection-steam oven (Rational Combi Steamer, Rational AG, Landsberg am Lech, Germany).
• Conditioning: After baking, the cookies were conditioned at 22 °C for 30 min before further analyses.

### 2.2. Sensory Evaluation

Sensory evaluation was performed using a 9-point hedonic scale to assess the sensory quality of the cookies [16]. A total of 120 consumers participated in the study and evaluated the samples for five sensory attributes: colour, taste, odor, crispness, and overall acceptability. A total of 120 adult consumers participated in the sensory evaluation. The participants constituted an untrained consumer panel and did not receive formal sensory training prior to the evaluation. They were regular consumers of bakery products and were recruited among students and staff of Wrocław University of Environmental and Life Sciences. A 9-point hedonic scale was used to assess consumer liking of the cookie samples. This scale was selected because it is a well-established method for measuring consumer acceptance of food products, particularly in studies involving untrained consumers [16]. The assessment was conducted in 12 sessions, with 10 consumers participating in each session. In each session, all cookie formulations were evaluated by the participants. Samples were coded with random three-digit numbers and presented in a randomized order to minimize potential order effects. Participants were provided with room-temperature water for palate cleansing between samples. The participants recorded their evaluations using specially designed questionnaires, with scores ranging from 1 (“dislike extremely”) to 9 (“like extremely”).

The sensory evaluation was conducted under controlled laboratory conditions, free from external factors that could influence sensory perception. All participants provided written informed consent before participation and were informed of their right to withdraw from the study at any time. The study was conducted in accordance with the principles of the Declaration of Helsinki [17]. The study protocol was approved by the Research Ethics Committee of Wrocław University of Environmental and Life Sciences (approval No. 28/2023). Personal data were anonymised and processed in accordance with the General Data Protection Regulation (GDPR 679/2016).

Based on the sensory evaluation results, cookie variants containing 0%, 10%, and 20% nettle powder and sweetened with sucrose (SU0, SU10, SU20) or erythritol (EU0, EU10, EU20) were selected for subsequent in vivo determination of glycaemic index.

### 2.3. Calculation of Nutritional Composition

The estimated nutritional composition of each cookie variant was calculated based on the nutritional declarations provided by the manufacturers of the individual ingredients. The calculated parameters included protein, fat, total carbohydrates, dietary fibre, available carbohydrates and energy. No direct proximate analysis of the final cookie products was performed. The estimated nutritional composition per 100 g of the experimental cookies is presented in Table 2.

### 2.4. Determination of Glycaemic Index and Glycaemic Load (In Vivo Study)

The study was conducted in accordance with the FAO/WHO guidelines [18] and the ISO 26642:2010 standard [19].

#### 2.4.1. Study Participants

Among the 34 individuals who volunteered for the study, 22 participants were ultimately included. The remaining 12 individuals were excluded based on the predefined eligibility criteria due to one or more of the following: abnormal fasting blood glucose levels, tobacco use, competitive/high-level sports activity, and/or insulin resistance. The final sample size was not determined by an a priori power analysis or formal effect-size calculation. Participants were recruited based on the predefined eligibility criteria, and the final number of 22 eligible participants exceeded the minimum number of participants recommended by ISO 26642:2010 for GI determination. The participants were aged 18–40 years (mean age: 23.0 ± 1.3 years) and had a normal body mass index (BMI: 18.5–24.9 kg/m^2^; mean BMI: 20.1 ± 1.78 kg/m^2^). The inclusion criteria were as follows: absence of diagnosed chronic diseases, particularly those affecting carbohydrate metabolism; no use of medications that could influence glucose metabolism; normal fasting blood glucose levels (≤99.0 mg/dL); no use of tobacco or psychoactive substances; moderate physical activity; and a standard diet.

Before the commencement of the study, each participant completed a detailed questionnaire regarding health status, dietary habits, and lifestyle. All participants were informed about the study objectives and procedures and received written information regarding their right to withdraw from the study at any time without providing a reason. Written informed consent was obtained from all participants prior to testing. The study was conducted in accordance with the Declaration of Helsinki [17]. The study protocol was approved by the Bioethics Committee of Wrocław Medical University (approval No. KB-848/2021).

#### 2.4.2. In Vivo Glycaemic Response Assessment

To standardise the glycaemic response, blood glucose concentration was measured after consumption of a reference solution containing 25 g of anhydrous glucose dissolved in 250 mL of water. The reference test was performed at the beginning and at the end of the study. Blood glucose concentration was measured once after consumption of each cookie variant. In accordance with the standardized methodology for GI determination, each test portion contained 25 g of available carbohydrates. Because the reformulation of the cookies with nettle powder and erythritol resulted in different amounts of available carbohydrates per 100 g of product, the physical mass of the portions necessarily differed between formulations. The portion sizes were therefore 54 g for SU0, 57 g for SU10, 60 g for SU20, 79 g for EU0, 86 g for EU10, and 93 g for EU20. These portions were used specifically for standardized GI determination and should not be interpreted as typical serving sizes. To provide additional information on practical dietary exposure, GL was subsequently calculated for a standardized 30 g serving of each cookie variant. The GI assessment was conducted in two testing sessions, with 11 participants tested per session. All test procedures were performed under the same standardized conditions, and the cookie formulations were evaluated according to the protocol described above. The test sessions were conducted at one-week intervals to provide an adequate wash-out period between consecutive product evaluations.

Capillary blood samples were collected from the fingertip in the fasting state and at 15, 30, 45, 60, 90, and 120 min after the beginning of cookie consumption using an Accu-Chek Softclix lancing device (Roche Diagnostics, Rotkreuz, Switzerland). Blood glucose concentrations were determined using an Accu-Chek Active glucometer (Roche Diagnostics, Rotkreuz, Switzerland).

The GI value of each cookie variant was calculated by dividing the incremental area under the blood glucose response curve (iAUC) after consumption of the test product by the iAUC obtained after consumption of the reference glucose solution and multiplying the result by 100. The mean iAUC values (mean ± SD) obtained for the reference glucose solution and individual cookie formulations are provided in Supplementary Table S1. Glycaemic load was calculated by multiplying the GI value by the amount of available carbohydrates in the consumed portion and dividing the result by 100. Individual GI and GL values were calculated for each participant, and the final values were expressed as arithmetic means. Values exceeding the mean by more than two standard deviations were excluded from the final analysis.

### 2.5. Statistical Analysis

Statistical analysis was performed using Statistica 13.3 PL software (StatSoft, Tulsa, OK, USA). The normality of data distribution was assessed using the Shapiro–Wilk test. Since the variables showed a normal distribution, one-way analysis of variance (ANOVA) was performed. Differences between mean values were evaluated using Tukey’s HSD post hoc test. Additionally, two-way ANOVA was conducted to determine the interaction effect between the percentage of nettle powder addition and the type of sweetener used. Statistical significance was set at *p* < 0.05.

## 3. Results

### 3.1. Sensory Evaluation

**Figure 1:**
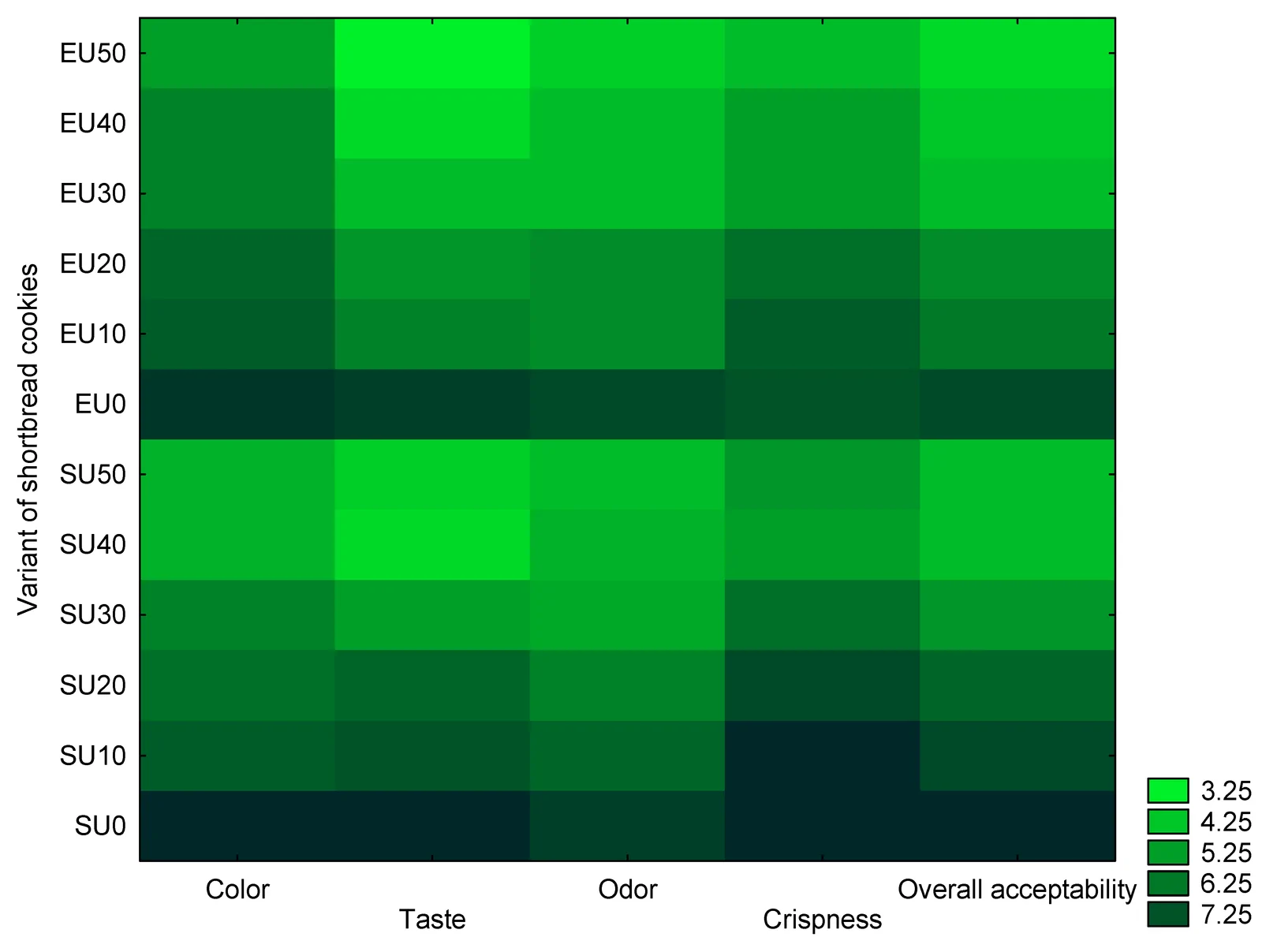
Sensory evaluation of shortbread cookies enriched with nettle powder. SU—sucrose-sweetened shortbread cookies with nettle powder; EU—erythritol-sweetened shortbread cookies with nettle powder; 0, 10, 20, 30, 40, and 50—percentage of wheat flour replacement with nettle powder (*w*/*w*). For exact numerical mean values, standard deviations, and statistical significance analysis, see Supplementary Table S2.

The results of the sensory evaluation of the analysed cookie variants are presented in Figure 1. Five sensory attributes were assessed: colour, taste, odor, crispness, and overall acceptability.

Colour acceptance was highest for the control cookies and progressively decreased with increasing nettle powder incorporation. The lowest colour scores were observed in cookies containing 50% nettle powder. A similar decreasing trend was observed for taste and odor, with the most pronounced reductions occurring at higher levels of nettle incorporation. Sucrose-sweetened cookies generally received slightly higher taste and overall acceptability scores than erythritol-sweetened variants, particularly at lower nettle powder levels.

Crispness remained relatively high in cookies containing up to 20% nettle powder but decreased markedly at higher substitution levels. Overall acceptability followed a similar pattern to the individual sensory attributes, with the highest scores observed for the control formulations and a progressive decline with increasing nettle addition, particularly above 20%. Nettle powder addition significantly affected the sensory characteristics of the cookies. Variants containing up to 20% nettle powder showed the highest sensory acceptance and were therefore selected for further analyses, including in vivo determination of glycaemic index.

### 3.2. Glycaemic Index and Glycaemic Load of Nettle-Enriched Shortbread Cookies

It should be noted that the physical mass of the test portions varied considerably between formulations because each portion was adjusted to provide the same 25 g of available carbohydrates. Therefore, the GI results should not be interpreted as representing the glycaemic effect of an equivalent mass or typical serving of each cookie. The use of carbohydrate-standardized portions allows comparison of carbohydrate quality between formulations, whereas the GL values calculated for a 30 g serving provide complementary information regarding the expected glycaemic impact of a realistic portion size.

#### 3.2.1. Glycaemic Index

**Figure 2:**
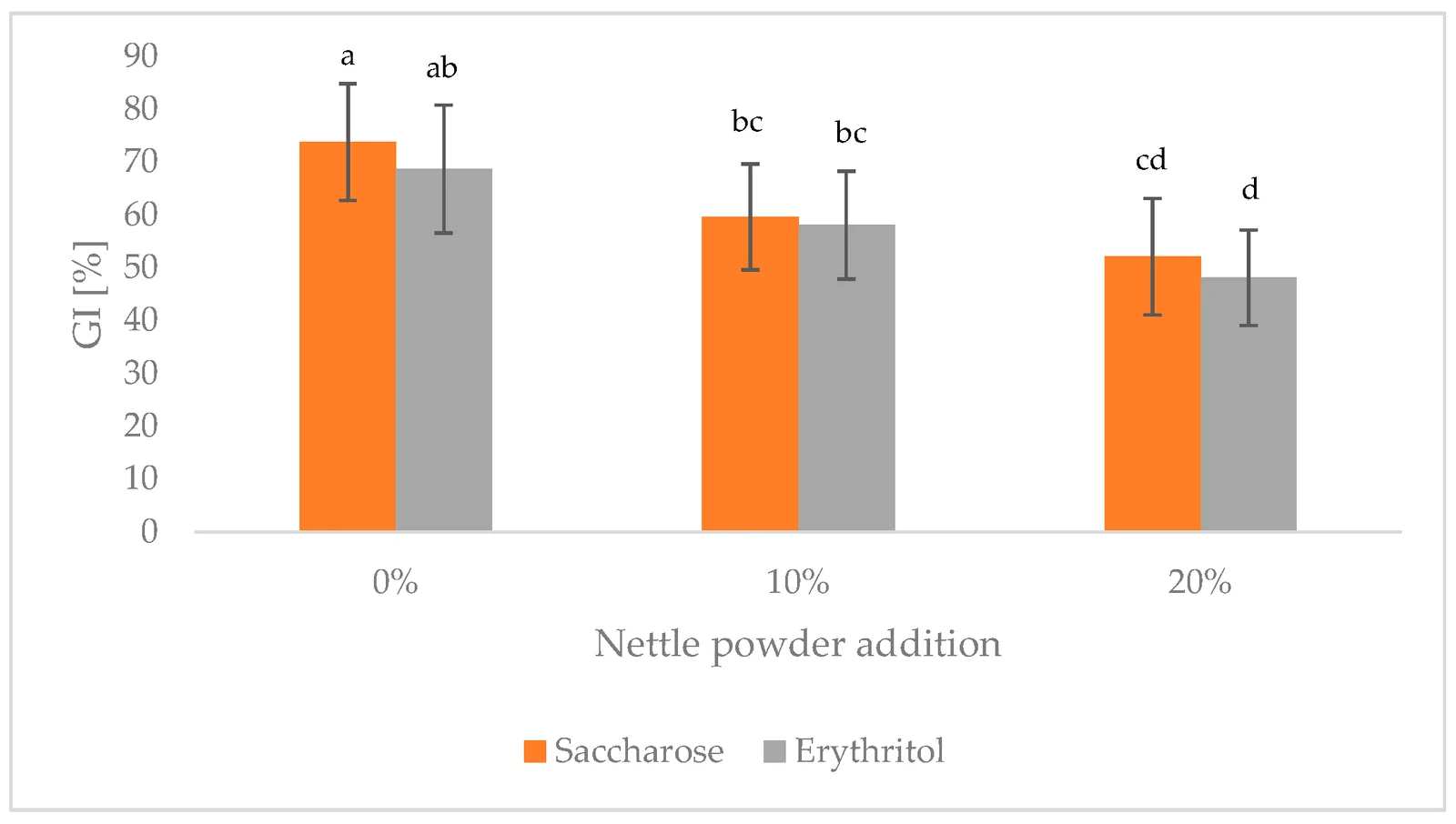
Glycaemic index of shortbread cookies enriched with nettle powder. Values with different superscript letters indicate significant differences among cookie formulations (*p* < 0.05; Tukey’s HSD test).

The glycaemic index (GI) values of the analysed shortbread cookies ranged from 48.10 to 73.77 (Figure 2). GI was determined for portions containing 25 g of available carbohydrates; therefore, the assessment reflected the carbohydrate quality of the products rather than the effect of a standard consumed portion.

In sucrose-sweetened cookies, increasing nettle powder incorporation significantly reduced GI values (*p* = 0.001), from 73.77 ± 11.05 in the control formulation to 52.10 ± 11.02 at 20% nettle powder addition. A similar significant reduction was observed in erythritol-sweetened cookies (*p* = 0.008), with GI decreasing from 68.69 ± 12.10 to 48.10 ± 9.03. Comparison of cookies with the same nettle powder addition level showed no statistically significant differences in GI between sucrose- and erythritol-sweetened variants (*p* > 0.05 for all comparisons). However, this finding should be interpreted in the context of the standardized GI methodology, in which each cookie portion provided 25 g of available carbohydrates. Consequently, the GI assessment was designed primarily to compare carbohydrate quality between formulations rather than the effect of consuming an equivalent mass or typical serving of sucrose- versus erythritol-sweetened cookies. Therefore, the absence of a significant sweetener effect on GI should not be interpreted as evidence that sucrose and erythritol have equivalent effects under real-life serving conditions.

According to the international GI classification, erythritol-sweetened cookies containing 20% nettle powder were classified as a low-GI product (GI ≤ 55), whereas sucrose-sweetened control cookies without nettle powder showed a high-GI response (GI ≥ 70) [20].

#### 3.2.2. Glycaemic Load

**Figure 3:**
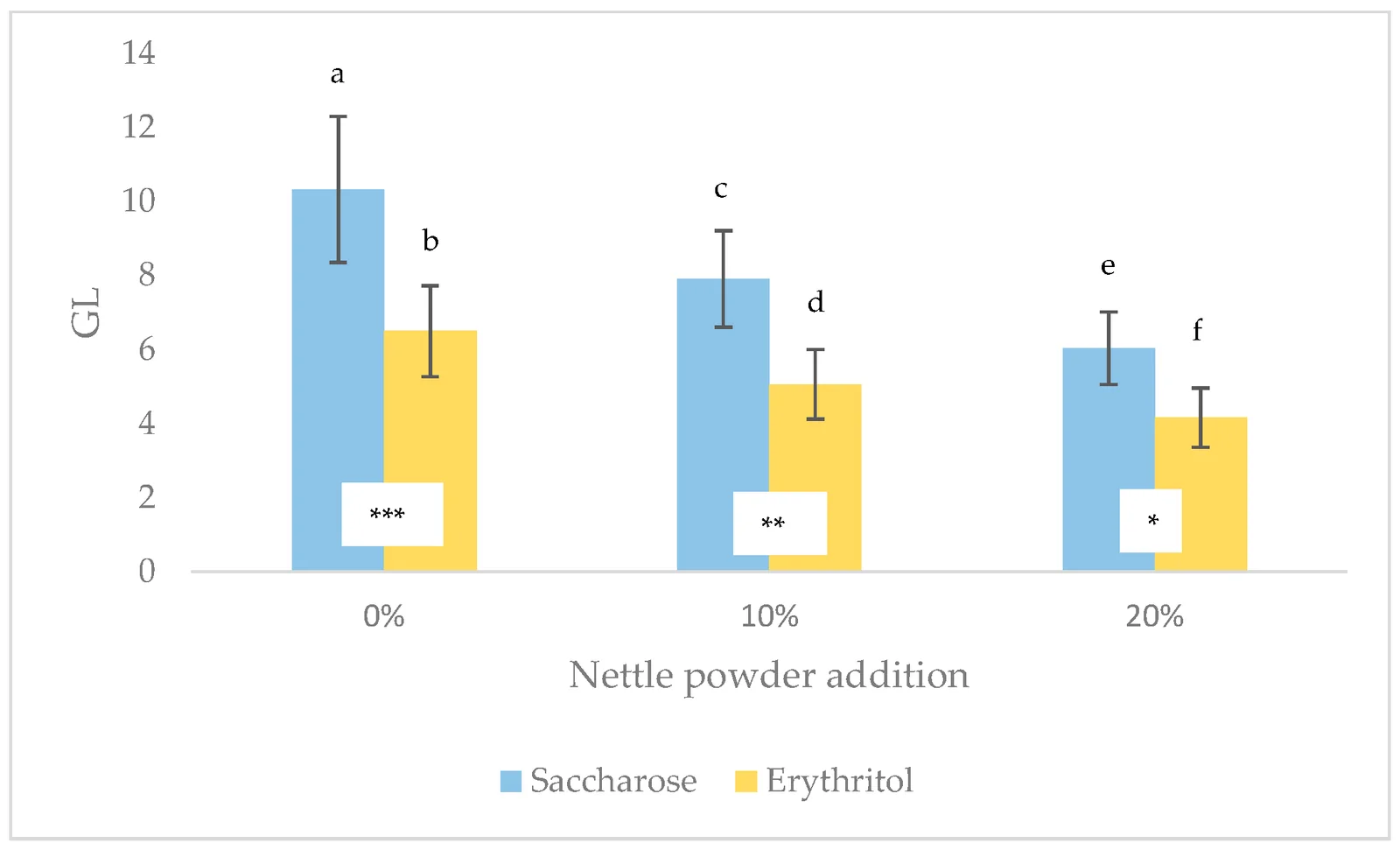
Glycaemic load of shortbread cookies enriched with nettle powder. Different superscript letters indicate significant differences among formulations (*p* < 0.05; Tukey’s HSD test). Asterisks indicate significant differences between sucrose- and erythritol-sweetened cookies at the same nettle powder addition level (* *p* < 0.05; ** *p* < 0.01; *** *p* < 0.001).

The glycaemic load (GL) values calculated for a standard serving size of 30 g are presented in Figure 3. Unlike GI determination, for which portions were adjusted to provide a fixed amount of 25 g of available carbohydrates, GL calculation was based on an identical physical serving size of 30 g for all cookie formulations. Therefore, GL provides complementary information on the potential glycaemic impact of consuming a realistic and directly comparable mass of the different products. The highest GL value was observed for sucrose-sweetened cookies without nettle powder addition (10.33 ± 1.97), whereas the lowest value was recorded for erythritol-sweetened cookies containing 20% nettle powder (4.16 ± 0.80).

In both sucrose- and erythritol-sweetened cookies, nettle powder addition significantly reduced GL values (*p* < 0.001 for both groups). The reduction in GL was proportional to the level of nettle powder incorporation. Moreover, regardless of the nettle powder addition level, erythritol-sweetened cookies showed significantly lower GL values compared with corresponding sucrose-sweetened variants (*p* < 0.05; Figure 3).

#### 3.2.3. Interaction Between Nettle Powder Addition and Sweetener Type

A two-way analysis of variance (ANOVA) was performed to evaluate the interaction between nettle powder addition level and sweetener type on GI and GL values (Table 3).

The main effect of nettle powder addition was significant for both GI and GL (*p* < 0.001 for both parameters). The main effect of sweetener type was significant for GL (*p* < 0.001), whereas no significant effect of sweetener type was observed for GI (*p* = 0.797). The absence of a significant sweetener effect on GI should be interpreted with caution because GI was determined using portions standardized to 25 g of available carbohydrates, which limits the assessment of the practical effect of replacing sucrose with erythritol on the glycaemic response of an equivalent serving size. In contrast, GL was calculated for a fixed 30 g serving and therefore provides a more direct indication of the effect of sweetener substitution on the glycaemic impact of a typical portion. No significant interaction was observed between nettle powder addition and sweetener type for either GI or GL (GI: *p* = 0.569; GL: *p* = 0.160).

**Table 3:** Two-way ANOVA results for the effects of nettle powder addition and sweetener type on glycaemic parameters.

Variable	Nettle Powder Addition (%)	Sweetener Type	Interaction (Nettle Powder × Sweetener Type)
GI	<0.001	0.797	0.569
GL	<0.001	<0.001	0.160

## 4. Discussion

### 4.1. Sensory Properties

The present study demonstrated that increasing the proportion of *Urtica dioica* powder progressively reduced the sensory acceptance of shortbread cookies, particularly with respect to colour, taste, odor and overall acceptability. The deterioration in sensory quality became especially evident when the substitution level exceeded 20%, whereas cookies containing 10 and 20% nettle remained well accepted by consumers. This indicates that moderate incorporation of nettle powder allows improvement of the estimated nutritional composition without markedly compromising consumer perception.

The reduction in colour acceptance with increasing nettle incorporation is consistent with the naturally dark green colour of nettle powder and its effect on the appearance of baked products. Similar decreases in colour acceptability have been reported for nettle-enriched bakery products [8]. Although chlorophyll degradation during baking may contribute to changes in colour, pigment composition was not analysed in the present study. Therefore, this mechanism should be considered a possible explanation rather than a directly demonstrated effect [21]. Taste and odor scores also decreased as the level of nettle increased. This effect is most likely related to the characteristic herbal taste profile of nettle, which may be associated with volatile compounds, chlorophyll derivatives and phenolic constituents naturally present in nettle leaves. Although these bioactive constituents contribute to the antioxidant potential of the product, they may also introduce grassy, earthy or slightly bitter notes that become increasingly perceptible at higher incorporation levels [5,6]. Similar sensory trends have been reported in nettle-enriched bakery products, where substitutions above approximately 20% resulted in significantly lower taste and overall acceptability despite improvements in nutritional composition [8]. These observations suggest that consumer acceptance of nettle-enriched bakery products is primarily limited by sensory rather than technological factors.

Texture is another critical determinant of cookie quality. In the present study, crispness remained relatively stable up to 20% nettle addition but decreased significantly at higher substitution levels. This observation may be related to the dilution of the gluten network following the replacement of wheat flour with plant material rich in dietary fibre. The presence of insoluble fibre particles may interfere with dough structure formation, alter water distribution and potentially affect the fracture properties of cookies [22,23,24]. However, crispness in the present study was assessed solely through sensory evaluation, and no instrumental texture analysis was performed. Therefore, these proposed structural effects should be interpreted cautiously, as they were not directly confirmed by instrumental measurements. Future studies incorporating instrumental texture analysis and structural characterisation of the cookie matrix would be valuable to verify these relationships.

Interestingly, cookies sweetened with sucrose generally received slightly higher sensory scores than those containing erythritol, particularly for taste and overall acceptability. Although erythritol provides approximately 60–80% of the sweetness of sucrose and possesses favourable technological properties, complete substitution of sucrose may alter taste perception, mouthfeel and texture because sucrose contributes not only sweetness but also bulk, caramelisation and taste development during baking [1,25,26,27]. Laguna et al. demonstrated that replacing sucrose with erythritol in short-dough cookies reduced consumer acceptance and crispness, particularly at higher substitution levels, despite maintaining acceptable technological properties [25]. Therefore, the slightly lower acceptance of erythritol-sweetened cookies observed in the present study appears consistent with previous findings and reflects the multifunctional technological role of sucrose in baked products.

The present results indicate a clear trade-off between increasing nettle powder incorporation and consumer sensory acceptance. Higher incorporation levels (30–50%) increased the contribution of nettle-derived nutrients and dietary fibre and were associated with a further reduction in available carbohydrates. However, these potential nutritional advantages were accompanied by a marked deterioration in colour, taste, odor, crispness and overall acceptability. In contrast, cookies containing 10–20% nettle powder maintained relatively high sensory acceptance while providing measurable nutritional and glycaemic benefits. Therefore, 10–20% nettle powder may be considered a practically suitable formulation range based on the combined sensory and nutritional results of the present study. This range should not be interpreted as a formally determined mathematical optimum, as a multi-objective sensory–nutritional optimization analysis was beyond the scope of the present investigation. Future studies should apply formal optimization approaches incorporating predefined sensory acceptance thresholds and nutritional targets to determine the optimal nettle incorporation level for specific product-development objectives.

### 4.2. Effect of Nettle on Glycaemic Index and Load

The present study demonstrated a significant reduction in GI and GL with increasing nettle powder incorporation. In sucrose-sweetened cookies, GI decreased from 73.77 in the control formulation to 52.10 at 20% nettle addition, while in erythritol-sweetened cookies it decreased from 68.69 to 48.10. These changes occurred alongside an increase in estimated dietary fibre content and a reduction in estimated available carbohydrate content (Table 2). The present study therefore demonstrates an association between nettle incorporation and a lower glycaemic response; however, the specific mechanisms responsible for this effect were not directly investigated. Accordingly, the mechanisms discussed below should be regarded as plausible explanations based on the existing literature rather than as experimentally confirmed mechanisms in the present study.

Several mechanisms may potentially contribute to the observed reduction in glycaemic response. The higher fibre content associated with nettle incorporation may reduce starch accessibility and slow carbohydrate digestion [28,29]. This interpretation is consistent with evidence from fibre-enriched cereal and bakery products, although the specific effects of nettle-derived fibre on starch digestion were not directly investigated in the present study [23,24,30]. Published analyses of Urtica dioica leaf material have reported crude fibre contents of approximately 9–11% on a dry-matter basis [30,31]. Thus, the contribution of nettle-derived fibre to the observed reduction in GI is biologically plausible. Nettle also contains phenolic compounds. Published analyses of nettle leaf powder have reported total phenolic contents of approximately 129 mg GAE/g on a dry basis [30], while studies using different extraction procedures have reported substantially lower values, illustrating the influence of extraction conditions and analytical methodology on the measured phenolic content [32]. Phenolic compounds may influence carbohydrate digestion through interactions with digestive enzymes or starch. For example, polyphenol-enriched cookies have been shown to exhibit increased inhibitory activity against α-amylase and α-glucosidase [21,33,34], while interactions between polyphenols and starch have also been described in modified starch systems [35]. However, the concentrations of individual phenolic compounds and their inhibitory activity were not measured in the present study.

The chlorophyll-rich composition of nettle may indirectly contribute to the observed reduction in GI. However, this potential relationship was not directly investigated in the present study. The effect may instead be related to the broader structural characteristics of the plant material, including its non-starch polysaccharides and cellular structures, which could limit enzyme accessibility to starch [36,37,38]. More generally, the incorporation of nettle powder may modify the food matrix by increasing fibre content and introducing structural barriers that affect starch accessibility and digestion [39]. These mechanisms remain hypothetical and require direct investigation in future studies.

The present findings are consistent with those reported by Tanyitiku et al. (2024), who observed a progressive decrease in the estimated glycaemic index of gluten-free corn cookies from approximately 49 to 33 following incorporation of 20% nettle leaf flour [8]. Although that study employed an in vitro digestion model, whereas the present investigation determined GI in vivo according to ISO 26642:2010, both studies demonstrated the same directional effect of nettle incorporation on carbohydrate digestibility. The agreement between in vitro and in vivo observations strengthens the evidence that nettle represents an effective functional ingredient for lowering the glycaemic potential of bakery products.

The reduction in GI and GL observed in the present study also agrees with previous investigations demonstrating that enrichment of cookies with fibre-and polyphenol-rich plant materials decreases starch digestibility and postprandial glycaemic response. Raczkowska et al. (2024) reported that combining erythritol with fruit pomace significantly reduced the in vivo glycaemic index of shortbread cookies, highlighting the importance of increasing dietary fibre and bioactive compounds within the bakery matrix [2].

From a product-development perspective, the reduction in glycaemic response should be considered together with the sensory results. Although increasing nettle incorporation was associated with progressively lower GI and GL, levels above 20% substantially reduced colour, taste, odor, crispness and overall acceptability. Thus, maximizing nettle incorporation would not necessarily result in the most suitable consumer product. The present findings suggest that moderate incorporation, particularly at 10–20%, may provide a more favourable balance between glycaemic properties and sensory acceptance. This balance is relevant for practical formulation decisions, although a formal multi-objective optimization considering nutritional, sensory, technological and economic parameters was beyond the scope of the present study.

### 4.3. Effect of Erythritol Substitution on Glycaemic Index and Load

The present study demonstrated that replacement of sucrose with erythritol significantly reduced the glycaemic load (GL) of shortbread cookies, regardless of the level of nettle addition. In contrast to the glycaemic index, which is calculated based on a standardised amount of available carbohydrates, GL reflects the actual glycaemic impact of a consumed portion and therefore better represents the physiological effect of a food product in real dietary conditions [40]. The lower GL observed in erythritol-containing cookies resulted primarily from the reduction in available carbohydrates and the negligible contribution of erythritol to postprandial glucose elevation. Erythritol is a polyol characterised by very low energy value and minimal metabolic utilisation. After ingestion, most erythritol is rapidly absorbed in the small intestine and excreted unchanged in urine, without undergoing significant metabolism to glucose. Consequently, erythritol is generally considered to have a negligible effect on blood glucose and insulin responses, which makes it a suitable sucrose substitute in products intended for individuals requiring glycaemic control [11,12].

The observed reduction in GL is consistent with previous studies investigating erythritol-containing bakery products. Raczkowska et al. (2024) demonstrated that replacement of sucrose with erythritol significantly reduced both glycaemic index and glycaemic load of shortbread cookies, particularly when erythritol was combined with fibre-rich plant ingredients [2]. Similar findings were reported by Laguna et al. (2013), who showed that erythritol can be successfully applied in cookie formulations; however, its technological functionality differs from sucrose due to differences in sweetness intensity, hygroscopicity and contribution to browning reactions [25]. Therefore, optimisation of erythritol concentration and use of complementary bulking ingredients may be necessary to maintain sensory properties comparable with sucrose-based products.

Although erythritol contributed substantially to the reduction in GL, its effect on GI could not be meaningfully distinguished under the carbohydrate-standardized conditions used for GI determination. Because all test portions provided 25 g of available carbohydrates, the GI assessment was not designed to capture differences in glycaemic impact associated with consuming an equivalent physical serving of sucrose- and erythritol-sweetened cookies. The significant reduction in GL observed for erythritol-sweetened cookies therefore provides more relevant information regarding the practical effect of sweetener substitution on the glycaemic impact of a defined serving size. This may be explained by the methodology used for GI determination, where products were consumed in portions containing an identical amount of available carbohydrates. Under these conditions, differences resulting from the type of sweetener are partially minimised because GI describes the quality of carbohydrates rather than the glycaemic effect of a realistic serving size. Therefore, the reduction in GL observed after erythritol substitution provides additional practical information regarding the nutritional value of the developed cookies.

### 4.4. Interaction Between Nettle Addition and Sweetener Type

The two-way ANOVA performed in the present study revealed no significant interaction between nettle addition and sweetener type for either GI or GL. Under the experimental conditions applied, this indicates that the effects of the two factors on the measured glycaemic parameters were statistically separable. However, the absence of a statistical interaction for GI and GL should not be interpreted as evidence of complete technological or practical independence of the two ingredients. In real-world product development, the combined incorporation of nettle powder and erythritol may influence processing behaviour and other product characteristics that were not evaluated in the present study.

The lack of interaction suggests that the mechanisms responsible for reducing GI and GL differed between the two factors. Nettle addition was associated with increased estimated dietary fibre content and may have influenced starch accessibility through changes in the food matrix and the presence of bioactive compounds, as suggested by previous research [7,21,36,37,38]. In contrast, erythritol primarily affected the carbohydrate composition of the product by replacing sucrose with a sweetener that contributes negligible available carbohydrates and has minimal metabolic impact [11,12].

Previous studies have shown that combining fibre-rich ingredients with sucrose reduction strategies can provide additive improvements in the nutritional quality of bakery products. Raczkowska et al. (2024) reported that the simultaneous use of erythritol and fruit pomace in cookies resulted in a lower glycaemic response; however, the contribution of each component was associated with different mechanisms [2]. The present study extends these observations by demonstrating that nettle powder and erythritol may be effectively combined to develop bakery products with reduced glycaemic impact without evidence of antagonistic effects.

From an industrial perspective, further evaluation is required before the combined formulation can be considered fully scalable. Nettle powder and erythritol may affect dough handling, cookie spread, colour development during baking, texture, moisture distribution and storage stability, particularly when used together. Economic considerations, including ingredient cost and availability, as well as regulatory and labelling requirements, may also influence the practical feasibility of the formulation. These parameters were outside the scope of the present study and should therefore be addressed in future technological and shelf-life investigations. Consequently, the present findings support the potential nutritional and glycaemic advantages of combining moderate nettle enrichment with erythritol, but do not establish technological independence or industrial scalability of the formulation.

### 4.5. Strengths and Limitations of the Study

The main strength of the present study was the comprehensive evaluation of the functional potential of nettle-enriched cookies by combining sensory assessment with in vivo determination of glycaemic response. In contrast to studies based solely on in vitro models of carbohydrate digestion, the present investigation evaluated glycaemic index according to internationally recognised methodology (ISO 26642:2010), providing more physiologically relevant information regarding the postprandial response to the developed products. An additional strength was the simultaneous application of two reformulation strategies: partial replacement of wheat flour with nettle powder and substitution of sucrose with erythritol. This experimental design enabled the evaluation of the individual effects of a plant-derived functional ingredient and a low-calorie sweetener, as well as the assessment of potential interactions between these factors. Furthermore, the inclusion of sensory evaluation involving 120 consumers provided valuable information regarding the practical acceptability and market potential of the developed cookies.

Several limitations of the study should also be considered. An important limitation of the study is that the in vivo glycaemic response assessment was conducted in a relatively small and homogeneous group of 22 healthy young adults with normal BMI. Therefore, the observed effects on GI and GL should be interpreted within the context of this specific population and should not be directly extrapolated to individuals with type 2 diabetes, insulin resistance, impaired glucose tolerance, older adults, or other populations with different metabolic characteristics. Further studies involving larger and more diverse populations, including individuals with impaired glucose metabolism, are needed to confirm the reproducibility and clinical relevance of these findings. Accordingly, the developed cookies should be considered a potentially lower-GI food option rather than a therapeutic product or an intervention for glycaemic control. Moreover, although nettle incorporation improved the glycaemic profile of the cookies, higher levels of substitution negatively influenced sensory acceptance, indicating the need for further optimisation of the formulation. Future studies should additionally investigate the detailed composition of bioactive compounds, antioxidant capacity, starch digestibility fractions and structural characteristics of the cookie matrix to better elucidate the mechanisms responsible for the observed effects. An additional limitation is that crispness was evaluated solely through sensory assessment, without instrumental texture analysis. Consequently, the potential effects of nettle powder on the structural and fracture properties of the cookies could not be objectively quantified. Future studies should combine sensory evaluation with instrumental texture measurements and structural characterisation of the cookie matrix to further elucidate the mechanisms underlying the observed changes in crispness. Despite these limitations, the results indicate that moderate incorporation of nettle powder combined with erythritol substitution may represent a promising approach for developing bakery products with improved nutritional properties and reduced glycaemic impact while maintaining satisfactory sensory quality.

An additional limitation is that the study did not include a formal multi-objective optimization procedure integrating sensory attributes and nutritional parameters. The identification of 10–20% nettle powder as the most suitable range was therefore based on the observed sensory acceptance together with the nutritional and glycaemic results rather than on a predefined optimization model. Such an approach could provide more precise formulation guidance for industrial product development and should be considered in future research.

## 5. Conclusions

The present study demonstrated that incorporation of Urtica dioica powder and replacement of sucrose with erythritol can modify the glycaemic properties of shortbread cookies while maintaining acceptable sensory quality at moderate nettle incorporation levels. Increasing the proportion of nettle powder significantly reduced glycaemic index and glycaemic load. These effects may be related to the higher dietary fibre content, the presence of bioactive plant compounds and changes in the food matrix that could affect starch accessibility. However, these mechanisms were not directly investigated in the present study and should therefore be considered proposed mechanisms.

Within the tested formulation range, cookies containing 10–20% nettle powder showed the most favourable combination of sensory acceptance and lower glycaemic impact. Higher nettle incorporation levels resulted in significant reductions in colour, taste, odor, crispness and overall acceptability, limiting their potential consumer acceptance. Erythritol substitution significantly reduced glycaemic load by lowering the amount of available carbohydrates in a typical serving, whereas its effect on glycaemic index was not significant under the applied GI testing conditions. The effects of nettle addition and sweetener type on glycaemic parameters did not show a significant interaction, indicating that the two factors contributed differently to the observed changes.

The combination of moderate nettle enrichment and erythritol substitution may therefore represent a promising formulation approach for developing shortbread cookies with lower glycaemic impact while retaining satisfactory sensory quality. However, the glycaemic effects observed in the present study were demonstrated in healthy young adults and should not be interpreted as evidence of therapeutic or clinical benefits. Further research involving larger and more diverse populations, as well as direct assessment of the proposed mechanisms, is required to determine the generalisability and clinical relevance of these findings. From an industrial perspective, future formulation work should consider the balance between nettle incorporation, erythritol substitution and sensory acceptance, together with technological properties, processing performance and product stability.

## Figures and Tables

**Table 1:** Formulation of the experimental shortbread cookies.

Ingredient	Nettle Powder Addition (%)					
	0%	10%	20%	30%	40%	50%
Wheat flour (type 450) [g]	300	270	240	210	180	150
Nettle powder [g]	0	30	60	90	120	150
Butter (82% fat) [g]	200					
Egg yolk [g]	60					
Sucrose/Erythritol [g]	100					

**Table 2:** Estimated nutritional composition of the experimental shortbread cookies based on manufacturers’ nutritional declarations (per 100 g).

Nettle Powder Addition (%)	Protein (g/100 g)	Fat (g/100 g)	Total Carbohydrates (g/100 g)	Dietary Fibre (g/100 g)	Available Carbohydrates (g/100 g)	Energy (kcal/100 g)
Sucrose-sweetened cookies						
0	6.71	27.96	48.05	1.36	46.69	472.00
10	7.41	28.04	46.19	1.95	44.24	467.36
20	8.10	28.12	44.33	2.54	41.79	462.73
Erythritol-sweetened cookies						
0	6.71	27.96	48.05	1.36	31.54	411.39
10	7.41	28.04	46.19	1.95	29.08	406.76
20	8.10	28.12	44.33	2.54	26.63	402.12

## Data Availability

The data supporting the findings of this study are contained within the article. The raw data underlying the results are available from the corresponding author upon reasonable request, subject to applicable ethical and privacy considerations.

## Supplementary Materials

The following supporting information can be downloaded at: https://www.mdpi.com/article/10.3390/nu18182973/s1, Table S1. Mean incremental area under the curve (iAUC) values for the reference glucose solution and nettle-enriched shortbread cookies; Table S2. Sensory evaluation scores of shortbread cookie variants (mean ± SD).

## References

[1] Pareyt B., Delcour J.A. (2008). The Role of Wheat Flour Constituents, Sugar, and Fat in Low Moisture Cereal Based Products: A Review on Sugar-Snap Cookies. Crit. Rev. Food Sci. Nutr..

[2] Raczkowska E., Bienkiewicz M., Gajda R. (2024). Modulation of the Glycaemic Index Value of Shortbread Cookies by the Use of Erythritol and Fruit Pomace. Sci. Rep..

[3] Doménech-Asensi G., Merola N., López-Fernández A., Ros-Berruezo G., Frontela-Saseta C. (2016). Influence of the Reformulation of Ingredients in Bakery Products on Healthy Characteristics and Acceptability of Consumers. Int. J. Food Sci. Nutr..

[4] Devkota H.P., Paudel K.R., Khanal S., Baral A., Panth N., Adhikari-Devkota A., Jha N.K., Das N., Singh S.K., Chellappan D.K. (2022). Stinging Nettle (*Urtica dioica* L.): Nutritional Composition, Bioactive Compounds, and Food Functional Properties. Molecules.

[5] Kregiel D., Pawlikowska E., Antolak H. (2018). *Urtica* spp.: Ordinary Plants with Extraordinary Properties. Molecules.

[6] Maietti A., Tedeschi P., Catani M., Stevanin C., Pasti L., Cavazzini A., Marchetti N. (2021). Nutrient Composition and Antioxidant Performances of Bread-Making Products Enriched with Stinging Nettle (*Urtica dioica*) Leaves. Foods.

[7] Krawęcka A., Sobota A., Pankiewicz U., Zielińska E., Zarzycki P. (2021). Stinging Nettle (*Urtica dioica* L.) as a Functional Component in Durum Wheat Pasta Production: Impact on Chemical Composition, In Vitro Glycemic Index, and Quality Properties. Molecules.

[8] Tanyitiku M.N., Bessem P., Petcheu I.C.N. (2024). Gluten-Free Corn Cookies Incorporated With Stinging Nettle Leaf Flour: Effect on Physical Properties, Storage Stability, and Health Benefits. Int. J. Food Sci..

[9] Fanzo J., McLaren R., Bellows A., Carducci B. (2023). Challenges and Opportunities for Increasing the Effectiveness of Food Reformulation and Fortification to Improve Dietary and Nutrition Outcomes. Food Policy.

[10] Huang Y., Chen Z., Chen B., Li J., Yuan X., Li J., Wang W., Dai T., Chen H., Wang Y. (2023). Dietary Sugar Consumption and Health: Umbrella Review. BMJ.

[11] Mazi T.A., Stanhope K.L. (2023). Erythritol: An In-Depth Discussion of Its Potential to Be a Beneficial Dietary Component. Nutrients.

[12] Younes M., Aquilina G., Castle L., Degen G., Engel K., Fowler P.J., Frutos Fernandez M.J., Fürst P., Gundert-Remy U., Gürtler R. (2023). Re-evaluation of Erythritol (E 968) as a Food Additive. EFSA J..

[13] Chiavaroli L., Lee D., Ahmed A., Cheung A., Khan T.A., Blanco S., Mejia, Mirrahimi A., Jenkins D.J.A., Livesey G. (2021). Effect of Low Glycaemic Index or Load Dietary Patterns on Glycaemic Control and Cardiometabolic Risk Factors in Diabetes: Systematic Review and Meta-Analysis of Randomised Controlled Trials. BMJ.

[14] Thomas D., Elliott E.J. (2009). Low Glycaemic Index, or Low Glycaemic Load, Diets for Diabetes Mellitus. Cochrane Database Syst. Rev..

[15] Reynolds A., Mann J., Cummings J., Winter N., Mete E., Te Morenga L. (2019). Carbohydrate Quality and Human Health: A Series of Systematic Reviews and Meta-Analyses. Lancet.

[16] Piggott J.R. (1988). Sensory Analysis of Foods.

[17] World Medical Association (2013). World Medical Association Declaration of Helsinki: Ethical principles for medical research involving human subjects. JAMA.

[18] FAO/WHO (1998). Carbohydrates in Human Nutrition. Report of a Joint FAO/WHO Expert Consultation (FAO Food and Nutrition Paper 66).

[19] (2010). Food Products—Determination of the Glycaemic Index (GI) and Recommendation for Food Classification.

[20] Atkinson F.S., Foster-Powell K., Brand-Miller J.C. (2008). International Tables of Glycemic Index and Glycemic Load Values: 2008. Diabetes Care.

[21] Pędziwiatr D., Lamadrid M.C., Wojdyło A. (2024). Cookies Fortified with Polyphenols Extracts: Impact on Phenolic Content, Antioxidant Activity, Inhibition of α-Amylase and α-Glucosidase Enzyme, Colour and Sensory Attractiveness. Antioxidants.

[22] Lesschaeve I., Noble A.C. (2005). Polyphenols: Factors Influencing Their Sensory Properties and Their Effects on Food and Beverage Preferences. Am. J. Clin. Nutr..

[23] Mancebo C.M., Rodríguez P., Martínez M.M., Gómez M. (2018). Effect of the Addition of Soluble (Nutriose, Inulin and Polydextrose) and Insoluble (Bamboo, Potato and Pea) Fibres on the Quality of Sugar-snap Cookies. Int. J. Food Sci. Technol..

[24] Blanco Canalis M.S., Steffolani M.E., León A.E., Ribotta P.D. (2017). Effect of Different Fibers on Dough Properties and Biscuit Quality. J. Sci. Food Agric..

[25] Laguna L., Primo-Martín C., Salvador A., Sanz T. (2013). Inulin and Erythritol As Sucrose Replacers in Short-dough Cookies: Sensory, Fracture, and Acoustic Properties. J. Food Sci..

[26] Dana H., Sonia A. (2024). Substituting Sugar in Pastry and Bakery Products with Functional Ingredients. Appl. Sci..

[27] Yan Z., Liu J., Cao S., Wang Z., Li C., Ren J., Zhang R., Zhang M., Liu X. (2023). Substitution of Sucrose by Erythritol in Angel Cake: Effect on Protein Foaming, Baking Performance and Digestion Properties. Int. J. Biol. Macromol..

[28] Qi X., Al-Ghazzewi F.H., Tester R.F. (2018). Dietary Fiber, Gastric Emptying, and Carbohydrate Digestion: A Mini-Review. Starch-Stärke.

[29] Tsitsou S., Athanasaki C., Dimitriadis G., Papakonstantinou E. (2023). Acute Effects of Dietary Fiber in Starchy Foods on Glycemic and Insulinemic Responses: A Systematic Review of Randomized Controlled Crossover Trials. Nutrients.

[30] Adhikari B.M., Bajracharya A., Shrestha A.K. (2016). Comparison of Nutritional Properties of Stinging Nettle (*Urtica dioica*) Flour with Wheat and Barley Flours. Food Sci. Nutr..

[31] Tarasevičienė Ž., Vitkauskaitė M., Paulauskienė A., Černiauskienė J. (2023). Wild Stinging Nettle (*Urtica dioica* L.) Leaves and Roots Chemical Composition and Phenols Extraction. Plants.

[32] Flórez M., Cazón P., Vázquez M. (2022). Antioxidant Extracts of Nettle (*Urtica dioica*) Leaves: Evaluation of Extraction Techniques and Solvents. Molecules.

[33] Ćorković I., Gašo-Sokač D., Pichler A., Šimunović J., Kopjar M. (2022). Dietary Polyphenols as Natural Inhibitors of α-Amylase and α-Glucosidase. Life.

[34] Melzig M.F. (2023). Plant Polyphenols as Inhibitors of Hydrolases Are Regulators of Digestion. Complement Med. Res..

[35] Ngo T.V., Kusumawardani S., Kunyanee K., Luangsakul N. (2022). Polyphenol-Modified Starches and Their Applications in the Food Industry: Recent Updates and Future Directions. Foods.

[36] Grundy M.M.-L., Edwards C.H., Mackie A.R., Gidley M.J., Butterworth P.J., Ellis P.R. (2016). Re-Evaluation of the Mechanisms of Dietary Fibre and Implications for Macronutrient Bioaccessibility, Digestion and Postprandial Metabolism. Br. J. Nutr..

[37] Tian J., Ogawa Y., Shi J., Chen S., Zhang H., Liu D., Ye X. (2019). The Microstructure of Starchy Food Modulates Its Digestibility. Crit. Rev. Food Sci. Nutr..

[38] Miao M., Hamaker B.R. (2021). Food Matrix Effects for Modulating Starch Bioavailability. Annu. Rev. Food Sci. Technol..

[39] Jia M., Yu Q., Chen J., He Z., Chen Y., Xie J., Nie S., Xie M. (2020). Physical Quality and in Vitro Starch Digestibility of Biscuits as Affected by Addition of Soluble Dietary Fiber from Defatted Rice Bran. Food Hydrocoll..

[40] Venn B.J., Green T.J. (2007). Glycemic Index and Glycemic Load: Measurement Issues and Their Effect on Diet–Disease Relationships. Eur. J. Clin. Nutr..

