# Supplementary material for: Functional Reformulation of Shortbread Cookies with Urtica dioica L. Powder and Erythritol: Effects on Sensory Quality, Glycaemic Index and Glycaemic Load
Source: Nutrients. 2026 Sep 11;18(18):2973. doi: 10.3390/nu18182973 (PMC13610514; doi:10.3390/nu18182973)
Supplement: Supplementary file 1 [file nutrients-18-02973-s001.zip › nutrients-4515295-supplementary.pdf]

Supplementary Table S1. Mean incremental area under the curve (iAUC) values for the reference glucose solution and nettle-enriched shortbread cookies,

Variant	iAUC – cookies (mean ± SD)
**Reference glucose**	2746.50 ± 552.18
**SU0**	2026.02 ± 60.99
**SU10**	1638.29 ± 55.23
**SU20**	1431.13 ± 60.85
**EU0**	1886.70 ± 66.80
**EU10**	1595.47 ± 56.31
**EU20**	1321.57 ± 49.86

SU: sucrose-sweetened shortbread cookies; EU: erythritol-sweetened shortbread cookies; 0, 10, 20, 30, 40, 50: level of wheat flour replacement with nettle powder (% w/w).

Supplementary Table S2. Sensory evaluation scores of shortbread cookie variants (mean ± SD)

Variant	Color	Taste	Odor	Crispness	Overall acceptability
**SU0**	8.15 ± 1.01 a	8.12 ± 1.15 a	7.72 ± 1.32 a	8.25 ± 0.86 a	8.17 ± 0.89 a
**SU10**	6.87 ± 1.80 bc	7.18 ± 1.85 ab	6.53 ± 1.68 bc	8.03 ± 1.01 ab	7.27 ± 1.44 ab
**SU20**	6.45 ± 1.86 cde	6.65 ± 1.58 bc	5.87 ± 1.66 cd	7.40 ± 1.38 abc	6.57 ± 1.60 bc
**SU30**	5.83 ± 2.16 def	5.10 ± 1.85 cd	4.98 ± 2.17 de	6.47 ± 1.83 bcd	5.50 ± 1.83 cd
**SU40**	4.72 ± 1.98 fg	3.70 ± 1.78 de	4.65 ± 1.90 e	5.13 ± 1.88 de	4.32 ± 1.63 de
**SU50**	4.53 ± 2.09 g	3.85 ± 1.92 de	4.42 ± 2.05 e	5.37 ± 2.15 cde	4.32 ± 1.80 de
**EU0**	7.95 ± 1.41 ab	7.62 ± 1.22 ab	7.48 ± 1.64 ab	7.18 ± 1.40 abc	7.50 ± 1.33 ab
**EU10**	7.00 ± 1.77 abc	5.98 ± 2.18 bc	5.73 ± 1.99 cd	6.77 ± 1.31 bc	6.18 ± 1.74 bcd
**EU20**	6.65 ± 1.74 bcd	5.38 ± 1.91 c	5.73 ± 1.81 cd	6.40 ± 1.56 bcd	5.70 ± 1.55 cd
**EU30**	5.97 ± 2.02 cde	4.27 ± 2.19 cde	4.42 ± 1.88 e	5.22 ± 1.82 cde	4.50 ± 1.77 de
**EU40**	5.65 ± 2.26 defg	3.62 ± 1.69 de	4.27 ± 2.02 e	5.12 ± 1.82 de	4.12 ± 1.54 de
**EU50**	5.05 ± 2.44 efg	3.03 ± 1.70 e	3.85 ± 2.14 e	4.50 ± 1.94 e	3.57 ± 1.48 e

SU: sucrose-sweetened shortbread cookies; EU: erythritol-sweetened shortbread cookies; 0, 10, 20, 30, 40, 50: level of wheat flour replacement with nettle powder (% w/w).
Values are expressed as mean ± standard deviation. Means in the same column followed by different superscript letters (a-g) are significantly different ($p < 0.05$) according to one-way ANOVA with Tukey's HSD post-hoc test.
